# Induction of Labour by High Puncture of the Membranes

**Published:** 1940

**Authors:** Coralie Rendle Short

**Affiliations:** Tutor in Obstetrics, University of Bristol; Senior Obstetric Officer, Southmead Hospital, Bristol


### INDUCTION OF LABOUR BY HIGH PUNCTURE OF THE MEMBRANES.

### A SURVEY OF EIGHTY-SEVEN CONSECUTIVE CASES PERSONALLY INDUCED DURING ONE YEAR—NOVEMBER, 1938—OCTOBER, 1939,

BY

CORALIE RENDLE SHORT, M.B., CH.B., M.R.C.O.G.,

*Tutor in Obstetrics, University of Bristol;*
*Senior Obstetric Officer, Southmead Hospital, Bristol.*

---

PREMATURE induction of labour has, during recent years, become a recognised treatment for many disorders of pregnancy, both obstetrical and medical. However, on studying the literature, it is found that there are several methods by which induction may be performed, some obviously more efficient and practicable than others.

Many American writers, such as Guttmacher and Douglas[1], Wilson[2], Morton[3], Slemans[4], Jackson[5] and Mathieu and Holman[6] prefer the combined technique of pituitary, castor oil, with or without quinine, together with rupture of membranes in front of the head. They vary a little in the order in which the drugs are given. The report of the Committee on Induction of Labour (1939)[7] showed that 100 out of 125 different physicians agreed with this; bags and bougies were generally condemned except in special cases. Wise,[8] in England, gives quinine for three weeks before rupturing the membranes. Pell[9] and Townend[10] use the bougie method of induction. Two writers, Drew-Smythe,[11], [12] who has a special cannula for the purpose, and Tennent[13] have used the high puncture method of rupturing the membranes.

It was found that out of about 2,000 deliveries, between November, 1938, and October, 1939, personally dealt with at St. James' Hospital, London, only 87 needed to be induced. Of these, 47 were primigravidæ and 40 multigravidæ. (A very few emergency cases of lateral placenta prævia, where the membranes were ruptured in front of the head, are not included.) The main indications were toxæmia, either severe or mild, certain medical conditions, and comparatively few for simple disproportion. This agrees with Tennent: of Drew-Smythe's cases the majority were for disproportion.

TABLE I. REASONS FOR INDUCTION. 87 CASES.

| | Primigravidæ 47. | Multigravidæ 40. |
|---|---|---|
| Mild or moderate toxæmia .. | 18 | 14 |
| Severe toxæmia or eclampsia .. | 5 | 6 |
| Toxæmia with disproportion | 10 | 4 |
| Toxæmia with twins .. .. | — | 1 |
| Ante-partum hæmorrhage .. | — | 3 |
| Post-maturity .. .. .. | 4 | 7 |
| Disproportion only .. .. | 6 | 4 |
| Pyelitis .. .. .. .. | 2 | — |
| Mitral stenosis .. .. .. | 1 | — |
| Insanity .. .. .. | 1 | — |
| Epilepsy .. .. .. | — | 1 |

NOTE.—Mild toxæmia means blood-pressure raised to about 150/90, and no albuminuria, or a trace of albumin in the urine with a blood-pressure above 125/75 after medical treatment: Severe toxæmia means blood-pressure over 180/110 and over two parts per thousand of albumen in the urine: Moderate toxæmia any condition between the two, where the patient did not improve on rest and diet.

Most of the patients were induced at thirty-nine weeks and onwards—none before thirty-six weeks.

TABLE II. TIME OF INDUCTION.

| Induced at. | Primigravidæ. | Multigravidæ. |
|---|---|---|
| 36—37 weeks .. .. .. | 6 | 8 |
| 38 weeks .. .. .. | 16 | 11 |
| 39 weeks onwards .. .. | 25 | 21 |

The method is quite simple and only on rare occasions was any difficulty encountered. In ordinary cases no anæsthetic was used, but in very nervous patients and in the severe toxæmic or eclampsia cases, a general anæsthetic was given. During the second half of the year, a combination of hyoscine gr. 1/100 with morphia gr. $\frac{1}{4}$ was given in the labour ward one hour before induction, and the patient left alone to sleep. This proved most successful and did not appear to have any ill effects on the mother or infant. In a few cases, however, the fœtal heart was a little irregular at the time of induction, but it always recovered and never gave rise to anxiety, nor did this account for any of the still-births.

TABLE III. ANÆSTHETIC USED.

| | Primigravidæ. | Multigravidæ. |
|---|---|---|
| None .. .. .. .. .. | 20 | 31 |
| General .. .. .. .. | 10 | 4 |
| Hyoscine and Morphine .. | 17 | 5 |

The patient was placed in lithotomy position, and the vulva and surrounding parts very carefully cleansed with dettol, and sterile towels arranged: a catheter was passed. Then a Sim's speculum was placed in the vagina, which again was washed out well with dettol. No volsellum was ever put on the cervix, and dilators were never necessary. A special point was always made of gently dilating the cervix with the finger at induction, and the membranes where possible were separated from it by running the finger round inside the internal os. The special catheter was then passed and the membranes punctured above the head; as a rule about fifteen to twenty ounces of liquor were drawn off. It was found that the latent period before labour started depended a great deal on the dilatation of the os, and hardly at all on the amount of liquor drawn off. In some cases labour started at once; in others, there was considerable delay, but in 64 per cent. of the primigravidæ and 70 per cent. of the multigravidæ, labour had started in less than twenty-four hours. These figures about correspond with those of Tennent, using the same method. If labour had not started in thirty hours, a full medical induction with quinine, castor oil, enema and pitocin was given. Wise and Wilson, who ruptured the membranes in front of the head and gave quinine as a routine, found the latent period under twenty-four hours in 80 to 90 per cent. of all cases, which is certainly quicker. However, we did not give quinine unless it was necessary because so many patients went into labour perfectly well without any further stimulation, and it is not an absolutely safe drug. Leslie Dodds[14] reported 1·45 per cent. fœtal deaths due to quinine alone in a series of medical inductions at the City of London Maternity Hospital. One interesting point was that, whereas in primigravidæ the earlier in pregnancy the induction was performed, the longer the latent period before labour started, in multigravidæ the average time of onset was about the same whether near term or not.

TABLE IV. LATENT PERIOD BEFORE LABOUR STARTED.

| | Primigravidæ. | Multigravidæ. |
|---|---|---|
| Under 6 hours.. .. .. | 18 (38.3%) | 18 (45%) |
| Under 12 hours .. .. | 23 (48.9%) | 25 (64.5%) |
| Under 24 hours .. .. | 30 (63.8%) | 28 (70%) |
| 24—48 hours .. .. .. | 9 (19.1%) | 7 (17.5%) |
| Over 48 hours .. .. .. | 7 (14.8%) | 5 (12.5%) |

TABLE V. AVERAGE TIME IN HOURS BEFORE LABOUR STARTED.

| Induced at. | Primigravidæ. | Multigravidæ. |
|---|---|---|
| 36—37 weeks .. .. .. | 25 hours 30 mins. | 17 hours 30 mins. |
| 39 weeks .. .. .. | 20 hours 30 mins. | 15 hours 30 mins. |
| 39 weeks onwards .. .. | 12 hours | 16 hours |

The average duration of labour (excluding three very abnormal cases of primary uterine inertia which would, if included, give a false impression) was 15 hours in primigravidæ and 8 hours 20 minutes in multigravidæ. This is almost identical with the figures given by Wise and very much shorter than in the bougie method of Peel (primigravidæ 32 hours 49 minutes, multigravidæ 18 hours 59 minutes). More interesting still is that labour was a good deal shorter than that given in the report on 14,396 normal non-induced cases at the John Hopkins Hospital, where the average time was 17·59 hours for primigravidæ and 11·75 hours for multigravidæ, showing that induction shortens rather than prolongs labour.

The number of spontaneous deliveries was 90·9 per cent., forceps being applied in 14·8 per cent. of the primigravidæ and 2·5 per cent. of the multigravidæ. In one case only the method failed, and the patient, a severe pre-eclamptic, got rapidly worse, so a lower segment Cæsarean section had to be performed; the mother and child both did well.

There were six still-births, three were in multigravidæ and three in primigravidæ. That is a gross total of 6·8 per cent. This figure at first looks rather high, but of the three primigravidæ, one death was due to eclampsia, one was a difficult forceps delivery of a ten-pound baby, and the third was an emergency case from another hospital who had had two previous quinine and pitocin inductions there. The multigravidæ comprised one case of severe accidental ante-partum hæmorrhage; one was the first twin in a severe toxæmia, and in one case only the cause was unknown and might possibly be due to the induction, so giving a corrected figure of two cases (one rather doubtful) or 2·3 per cent. still-births. It should also be taken into account that many of the cases were for toxæmia. Comparing this result with others, Tennent gives a corrected figure of 3·53 per cent., Wise of 3·1 per cent., and in the 14,396 normal cases at the John Hopkins Hospital the number given is 5·08 per cent. still-births. As one of the chief arguments against induction is that the still-birth rate is increased, we feel that considering the low figure actually recorded, and the number of potential still-births that might have occurred had the patient not been induced, induction is quite justified in carefully chosen cases. The neo-natal deaths were two (2·3 per cent.), both in multigravidæ; one was a case of severe toxæmia, the other followed an external version in a mentally deficient patient with severe epilepsy who had no idea of her " dates." Practically all the other babies did well, and were not unduly small.

Another main criticism against induction is that it causes puerperal sepsis. In this series, there was a gross total of 16 per cent. puerperal pyrexia, taking the standard as 99·6° F. (not 100·4°). The causes for the pyrexia are shown in Table VII. In two cases only was there no other cause except the induction (corrected total 2·3 per cent.) and neither of these cases was notifiable as the temperature did not go above 100·4° F.

TABLE VI. WEIGHTS OF THE BABIES AT BIRTH.

| Weight. | Primigravidæ. | Multigravidæ. |
|---|---|---|
| 3 lbs. .. .. .. .. | — | 3 |
| 4 " .. .. .. .. | 3 | 4 |
| 5 " .. .. .. .. | 6 | 4 |
| 6 " .. .. .. .. | 15 | 13 |
| 7 " .. .. .. .. | 17 | 2 |
| 8 " .. .. .. .. | 2 | 9 |
| 9 " .. .. .. .. | 2 | 2 |
| 10 " .. .. .. .. | 1 | 1 |
| | +1 unweighed | +2 unweighed |

TABLE VII. PUERPERAL PYREXIA (ABOVE 99.6° F.).

| Cause. | Primigravidæ 10. | Multigravidæ 4. |
|---|---|---|
| Urinary infection .. .. | 3 | 2 |
| Forceps deliveries .. .. | 4 | 1 |
| Cæsarean Section (stitch abscess) | 1 | — |
| Retained cotyledon .. .. | — | 1 |
| Induction the only interference | 2 | — |

There were no maternal deaths. In comparison with other writers, Tennent reports a sepsis rate of 3·08 per cent., with 2·8 per cent. maternal mortality: Drew-Smythe 3·9 per cent. morbidity with 1·95 per cent. mortality, both using the cannula method. Wise had two deaths and 2·7 per cent. notifiable pyrexia after rupturing the membranes in front of the head. Wilson, with the same method, had a 3·9 per cent. maternal mortality, and 7·6 per cent. pyrexia, whilst Peel, using bougies, had one death and 48·8 per cent. of his cases had a temperature over 99·6°. Against all this,

Mathieu and Holman in 750 non-induced cases, had a mortality of 0·27 per cent., and puerperal pyrexia 6·6 per cent.

Therefore it may be said that inducing labour by rupturing the membranes in selected cases is a commendable procedure. The high puncture method is simple to perform after a little practice and does not usually require an anæsthetic. Its only disadvantage is that labour may be longer in starting than when the membranes are ruptured in front of the head. It is important that the bladder should be empty and that the finger should guide the cannula all the time, otherwise it might theoretically be possible to damage the bladder, to push the cannula through the uterus or to dislodge a low-lying placenta. In one case only was there any considerable bleeding at the time of induction, but it soon stopped and neither the mother nor the infant came to any harm. Many writers point out the dangers of prolapse of the cord, but it never occurred in this series, probably because so few were done for disproportion. The method also was efficient : in only one case was there a failure. The mothers like it better than puncturing the membranes in front of the head because they are not always " wet " afterwards ; in most cases where the head is engaged in the pelvis there is practically no leakage of liquor. The length of labour is actually shorter than normal : it is not prolonged in any way. The amount of operative interference necessary is also much less in cases that are induced, and if Cæsarean section ever needs to be performed afterwards, provided the induction was carried out under strict aseptic conditions, the risk of sepsis should not be greatly increased. Probably it is less with high puncture than with low, because there is not likely to be ascending infection from the vagina.

The main objections to induction are that the maternal morbidity and mortality are increased because of the risk of sepsis, the still-birth rate is higher, and a lot of premature infants are born. It has been shown that this fear is ungrounded because, in this series, more than half (50·5 per cent.) were induced for toxæmia, and 12·5 per cent. of these had very severe toxæmia or even eclampsia, and would probably have had small babies anyway. The most debatable point is, however, whether or not induction should be performed in primigravidæ for pure disproportion with no other complication. Only six cases out of a total of two thousand deliveries in this hospital were so induced although Drew-Smythe recommends the practice.

No method of induction is perfect, but we did not find the sepsis or still-birth rate materially increased. The babies were nearly all a reasonable size, and they did well. On these grounds this method of induction is to be recommended.

I wish to express my thanks to Dr. Allen Daley, Chief Medical Officer of the London County Council for allowing this article to be published ; also to Mr. R. L. Dodds for his kind help and advice.

### REFERENCES.

[1] Guttmacher, A. E., and Douglas, R. E., *Amer. Jour. Obstet. and Gynæcol.*, 1931, xxi. 485.

[2] Wilson, L., *ibid.*, 1934, xxvii. 329.

[3] Morton, D. J., *ibid.*, 1933, xxvi. 323.

[4] Slemans, J. M., *ibid.*, April, 1932, xxiii. 492.

[5] Jackson, D. L., *Trans. Amer. Assn. Obstet., Gynæcol. and Abd. Surgery*, 1928, xli. 315.

[7] "Report of the Committee on Induction of Labour," *ibid.*, 1939, xxxvii. 873.

[8] Wise, E. C., *Brit. Med. Jour.*, 1939, i. 665.

[9] Peel, J. H., *Lancet*, 1936, i. 935.

[10] Townend, E. M., *Brit. Med. Jour.*, 1929, ii. 1197.

[11] Drew-Smythe, H. J., *ibid.*, 1931, i. 1018.

[12] Drew-Smythe, H. J., and Thompson, J., *Jour. Obstet. and Gynæcol. British Empire*, 1937, xliv. 480.

[13] Tennent, R. A., *ibid.*, 1938, xlv. 509.

[14] Dodds, R. L., *ibid.*, 1931, xxxviii. 827.

